# Success and Limitation of Equine Influenza Vaccination: The First Incursion in a Decade of a Florida Clade 1 Equine Influenza Virus that Shakes Protection Despite High Vaccine Coverage

**DOI:** 10.3390/vaccines7040174

**Published:** 2019-11-02

**Authors:** Stéphanie Fougerolle, Christine Fortier, Loïc Legrand, Marion Jourdan, Christel Marcillaud-Pitel, Stéphane Pronost, Romain Paillot

**Affiliations:** 1 Frank Duncombe, LABÉO, 14280 Saint Contest, France; st.fougerolle@gmail.com (S.F.); christine.fortier@laboratoire-labeo.fr (C.F.); loic.legrand@laboratoire-labeo.fr (L.L.); stephane.pronost@laboratoire-labeo.fr (S.P.); 2 NORMANDIE UNIV UNICAEN, Biotargen, 14000 Caen, France; 3 RESPE, 3 rue Nelson Mandela, 14280 Saint-Contest, France; technicien@respe.net (M.J.); c.marcillaud-pitel@respe.net (C.M.-P.)

**Keywords:** equine influenza virus, horse, vaccination, immune coverage, DIVA test, surveillance, epizooty, Florida Clade 1 (FC1)

## Abstract

Every year, several epizooties of equine influenza (EI) are reported worldwide. However, no EI case has been identified in France between 2015 and late 2018, despite an effective field surveillance of the pathogen and the disease. Vaccination against equine influenza virus (EIV) remains to this day one of the most effective methods to prevent or limit EI outbreaks and the lack of detection of the pathogen could be linked to vaccination coverage. The aim of this study was to evaluate EI immunity and vaccine coverage in France through a large-scale serological study. A total of 3004 archived surplus serums from French horses of all ages, breeds and sexes were selected from four different geographical regions and categories (i.e., sanitary check prior to exportation, sale, breeding protocol or illness diagnosis). EIV-specific antibody response was measured by single radial hemolysis (SRH) and an EIV-nucleoprotein (NP) ELISA (used as a DIVA test). Overall immunity coverage against EIV infection (i.e., titers induced by vaccination and/or natural infection above the clinical protection threshold) reached 87.6%. The EIV NP ELISA results showed that 83% of SRH positive serum samples from young horses (≤3 years old) did not have NP antibodies, which indicates that the SRH antibody response was likely induced by EI vaccination alone (the HA recombinant canarypoxvirus-based EI vaccine is mostly used in France) and supports the absence of EIV circulation in French horse populations between 2015 and late 2018, as reported by the French equine infectious diseases surveillance network (RESPE). Results from this study confirm a strong EI immunity in a large cohort of French horses, which provides an explanation to the lack of clinical EI in France in recent years and highlights the success of vaccination against this disease. However, such EI protection has been challenged since late 2018 by the incursion in the EU of a Florida Clade 1 sub-lineage EIV (undetected in France since 2009), which is also reported here.

## 1. Introduction

Equine influenza (EI) is one of the most important respiratory diseases of horses. Beyond the welfare issue induced by this infectious disease, the potential impact for the equine industry could be devastating, as clearly illustrated in 2007 when EI reached for the first time the naïve population of Australia, it infected over 76,000 horses [1]. The overall cost for the Australian economy to control this epizooty and to regain its EI-free OIE status was estimated to have reached A$1 billion.

Due to the density of the equine population and a very high transmission capacity, prevention methods such as vaccination are essential to prevent or control EI outbreaks. The efficacy of vaccines against equine influenza virus (EIV) infection has been demonstrated through numerous clinical and field studies [2,3,4,5] but little or no information is available about EI immunity in the field and vaccine coverage in horse populations. In humans, most of the studies that investigate vaccination coverage are based on an epidemiological questionnaire and showed that the influenza vaccination coverage is often less than 60% [6,7,8,9]. As for equine influenza, vaccine coverage is well known to be essential to prevent human influenza, as recently illustrated by Uchida et al. [10], who showed that high vaccine coverage was significantly and negatively correlated with the level of influenza epidemic among elementary school units. A postal questionnaire survey of randomly selected horse owners in Great Britain has reported a frequency of vaccination against EI and tetanus of 71.3% [11]. Through a retrospective mathematical modeling study of the 1971 Japanese EI outbreak in racehorse facilities, Satou and Nishiura calculated that 50% to 80% of an equine population should be vaccinated with a completely effective EI vaccine to achieve protection against an EIV strain with a relatively low to moderate rate of transmission, respectively. Taking into account that sterilizing immunity is rarely achieved by EI immunization and that EI vaccines efficacy wanes with time, the author also provided a more realistic vaccine coverage threshold of 86.5% [12].

The French horse population regroups 1.106 million horses (as recorded in 2016), which represents 15% of the European equid population with France placed third in terms of number behind Germany and the United Kingdom. Despite active disease surveillance that involves around 800 equine veterinary practitioners taking part in the French equine pathology epidemiological surveillance network (RESPE), EI has not been detected in France between mid-2015 to early December 2018 [13]. During this period, there was evidence of EIV circulation in other European countries and worldwide [14,15,16,17]. Since early December 2018, several European countries (Belgium, France, Germany, Ireland, the Netherlands, Sweden and the United Kingdom) have reported unusual levels of EIV circulation. The number of EI outbreaks is staggering in some countries, with more than 200 outbreaks reported in the United Kingdom between January and October 2019, leading to a 6 day shutdown of horse racing in February (the last shutdown linked to EI dated from 1979) and more than 174 racing stables placed in lockdown with mandatory EIV testing. This situation, unseen in Europe since the late 1970s and 1980s, reminds us of the impact of the 5 months long EI in Australia in 2007, when over 76,000 horses were infected with EIV and with an economical cost reaching A$1 billion [1]. The H3N8 EIV at the origin of the current European outbreaks belongs to the Florida Clade 1 (FC1) sub-lineage, which was usually circulating in North and South Americas [17,18,19,20] with only occasional appearances in Europe from time to time [18]. FC1 EIV strain was not isolated in France since 2009 [13].

The current report aims to present and discuss the results from a large-scale sero-epidemiological study designed to provide a picture of the immunological status against EI of the French horse population in late 2017 and the first incursion in a decade of an FC1 EIV. These results should provide information about vaccine coverage and an explanation about the absence of reported EI outbreaks between mid-2015 and late 2018. Unlike previous studies on vaccination coverage against human and equine influenza A viruses, this study is based on an evaluation of antibody levels measured by single radial hemolysis assay (SRH), a well-recognized correlate of protection against EIV infection and by an enzyme-linked immunosorbent assay (ELISA), which detects antibodies against the viral nucleoprotein (NP) of type A influenza viruses and could be used as a DIVA (differentiating infected from vaccinated animals) marker in specific situations [1]. Due to the large-scale EI epizooty affecting France and other European countries since late 2018/early 2019, details of the French EI outbreaks will also be reported. A phylogenetic analysis of the EIV strains involved and the detail of amino acid substitutions in the hemagglutinin (HA) protein sequence and antigenic sites [15,18], which are primary targets for virus-neutralizing antibody response, will be presented. These elements will be discussed in relation to EI vaccination in the field and the risk of vaccine breakdown.

## 2. Materials and Methods

### 2.1. Serum Samples

The serological study was carried out on archived surplus serum samples from the LABÉO Diagnostic and Research Institute. The initial serum samples were obtained, for diagnostic reasons unrelated to this study, from 3004 individual horses located in France, of all ages, breeds, sexes and born in different countries over two time periods, from June 2017 to March 2018 (time period 1, *n* = 2645) and from December 2018 to April 2019 (time period 2, *n* = 359). All serum were kept at −20 °C until analysis. Archived sera were randomly selected from 4 different geographical regions in France with some of the highest equids and breeding center densities (Normandy, *n* = 1837; Pays de la Loire, *n* = 269; Auvergne-Rhône-Alpes, *n* = 240 and Occitanie, *n* = 299) and taking into account the North–South and East–West localization (Supplementary Figure S1). The larger number of samples from Normandy is explained by the highest number of horses and breeding centers in this specific region and the location of the LABÉO Institute. Samples were subdivided into 4 categories according to the initial diagnostic analyses requested: sanitary check for exportation, sale, breeding or illness diagnosis. Samples from late 2018 to early 2019 came from the north of France, including Normandy, where EI outbreaks were reported at the time of sampling (cf. Section 3.4). The number of serum samples per region and categories is detailed in the Supplementary Table S1. In France, EI vaccination is mandatory for some of these categories (exportation, sale and breeding) but the vaccination history of these samples was unknown. Samples were anonymized by a third party prior to analysis, with the age of the horse at the time of sampling and its country of origin, (i.e., France or others) being the only information available for this study. This work received ethical approval from the LABÉO ethical advisor. During the 2018–2019 EI epizooties, 11 serum samples were obtained from field veterinary practitioners in the context of the epidemiological investigation conducted by the RESPE. 

### 2.2. Single Radial Haemolysis (SRH)

Antibodies were measured by single radial hemolysis (SRH) assay against the EIV strain A/equine/Jouars/4/06 (H3N8; Florida Clade 2), as previously described [16]. A control reference serum (A/equine/South Africa/4/03; H3N8; Florida Clade 1; reference Eu SA/4/03 Y0000712) from the European Directorate for the Quality of Medicines and Healthcare (EDQM) was included on each plate and used to standardize the results [21]. The titers of SRH antibody were expressed as the area of hemolysis (mm^2^). SRH antibody titers measured are correlated to protection (when there is no significant mismatch between the EIV vaccine and circulating strains); clinical signs of EI are significantly reduced when SRH antibody levels reach 85 mm^2^ or greater, virus shedding is significantly reduced with SRH antibody titer of 154 mm^2^ or greater [22,23].

### 2.3. EIV NP ELISA (DIVA Test)

The competition ID Screen Influenza A Antibody Competition Multispecies ELISA for the detection of anti-nucleoprotein (NP) antibodies of the Influenza A virus was carried out in accordance with the manufacturer’s instructions (ID Vet Innovative Diagnostics, Grabels, France). Briefly, the results were measured and recorded at the optical density (OD) at 450 nm using Thermo Labsystems Opsys MR (Fisher Scientific, Hampton, NH, USA). For each sample, the competition percentage was calculated as follows: (sample OD value/negative serum control OD value × 100). The samples with a competition percentage of 45% or less were defined as positive and the samples with a competition percentage of 50% or more were considered as negative. A total of 495 serum samples were analyzed (410 for the time period 1 and 85 for the time period 2). 

### 2.4. EIV Genes Sequencing and Phylogenetic Analysis

The HA and neuraminidase (NA) sequences from 2018/2019 French EIV outbreak isolates were determined. The phylogenetic analysis was carried out for HA1 only. Gene was amplified in one-step after extraction. PCR products were generated using SuperScript III One-Step RT-PCR System with Platinum Taq High Fidelity (Invitrogen). HA and NA were amplified with M13 primer sequences as described previously [24]. Sequencing was performed by Biofidal (Vaulx en Velin, France) using the Sanger method. Sequences were assembled and contigs were analyzed with the CodonCode Aligner v1.5.2 software (CodonCode Corporation, Dedham, MA, USA). Multiple alignments of all sequences were conducted using the Muscle algorithm and neighbor-joining trees method (MEGA V7 software version 7.2.26, Pennsylvania State University, University Park, PA, USA [25]), with a maximum likelihood substitution model and bootstrapped 1000 times to assess the reliability.

### 2.5. Statistical Analysis

Statistical analysis was performed using R version 3.5.1 (R foundation for statistical computing, Vienna, Austria). Statistical significance was based on a Chi-Square test (contingency table with 2 to 4 categories as columns and the 4 levels of SRH titers as lines). The level of significance was set as *p*-value < 0.05.

## 3. Results

### 3.1. Overall SRH Antibody Response and Correlation with EI Immune Status

**Figure 1:**
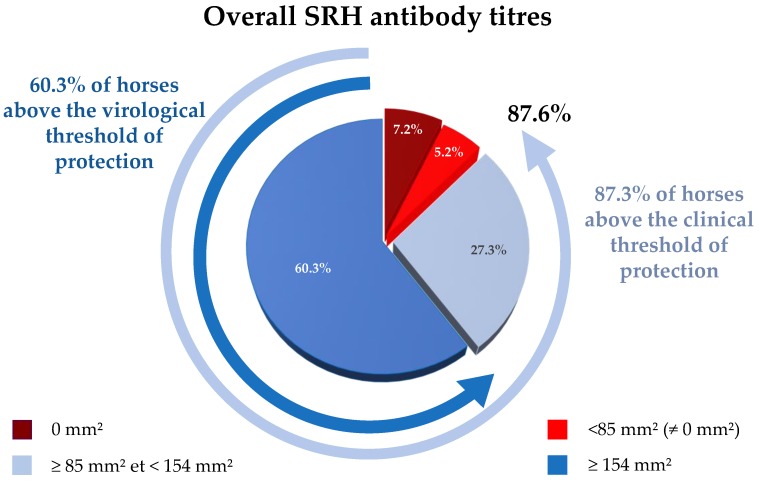
Overall single radial hemolysis (SRH) antibody response for serum samples collected between 2017 and 2018 (all four French regions combined). SRH titers below the clinical protection threshold (>85 mm^2^) are in red colors, titers above the clinical protection threshold are in light blue (85 mm^2^ and <154 mm^2^), titers above the clinical and virus shedding protection threshold (154 mm^2^ and above) are in blue.

The SRH antibody titers of the 2645 serum samples analyzed are summarized in Figure 1 and Supplementary Table S1. Of these samples, 12.4% (*n* = 328) were below 85 mm^2^ (i.e., horses considered to be at risk of infection), including 190 samples with SRH antibody titer = 0 mm^2^ and 138 seropositive samples (57.5 ± 17.4 mm^2^). Of remaining samples, 27.3% were ≥85 mm^2^ and <154 mm^2^ (128.1 ± 19.1 mm^2^; *n* = 721; i.e., horses considered as clinically protected) and 60.3% ≥ 154 mm^2^ (194.8 ± 28 mm^2^; *n* = 1596; i.e., horses considered as optimally immunised with clinical protection and little or no virus shedding in case of EIV infection). Overall, 87.6% of serum samples tested possessed SRH antibody titers above the clinical protection threshold (85 mm^2^).

### 3.2. SRH Antibody Analysis by Geographic Region and Category

**Figure 2:**
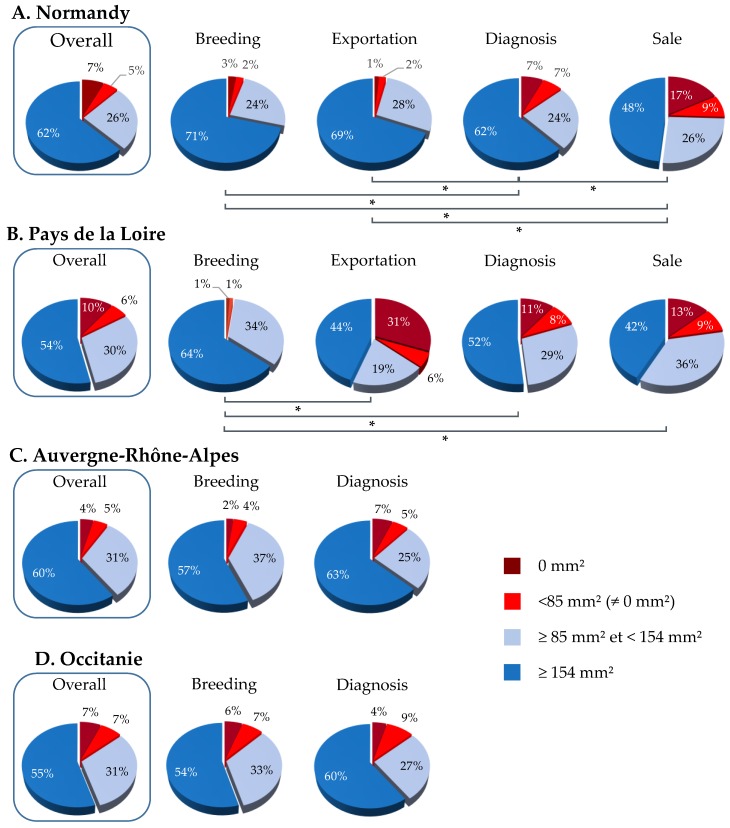
SRH antibody responses obtained from different geographical regions in France. Overall and categories analysis of immune coverage for Normandy (**A**), for Pays de la Loire (**B**), for Auvergne-Rhône-Alpes (**C**) and for Occitanie (**D**). SRH titers below the clinical protection threshold (>85 mm^2^) are in red colors, titers above the clinical protection threshold are in light blue (85 mm^2^ and >154 mm^2^), titers above the clinical and virus shedding protection threshold (154 mm^2^ and above) are in blue. (✻) *p*-value ≤ 0.0001.

**Figure 3:**
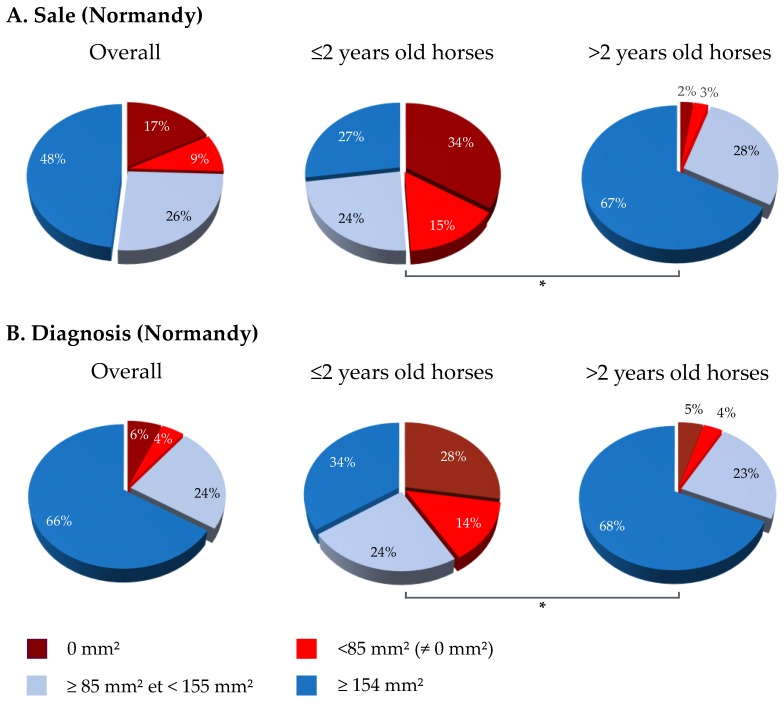
Analysis of “Sale” and “Diagnosis” categories in Normandy. SRH antibody response in ≤2 and >2 years old horses at the time of sample collection (horses born in 2016 and 2017). (**A**) Sale category and (**B**) Diagnosis category. (✻) *p*-value ≤ 0.00001.

In Normandy, 87.9% of serum samples were above 85 mm^2^ (175.9 mm^2^; *n* = 1614; Figure 2), including 62.3% of samples ≥154 mm^2^ (195.2 ± 28.3 mm^2^; *n* = 1144). The SRH antibody analysis of 1837 serum samples by categories showed that 74.4%, 95.2%, 96.4% and 86.5% of samples were ≥85 mm^2^ for the sale, breeding, exportation and diagnosis categories, respectively (Figure 2A), with a statistically significant difference between categories (*p*-value < 0.00001). The frequency of serum samples reaching at least the clinical protection threshold for the sale category was significantly lower than that of other categories (breeding, exportation and diagnosis; *p*-values ≤ 0.00001, 0.00001 and <0.0001, respectively). After the exclusion of samples from horses born in 2016 and 2017 for the sale category, the frequency of serum samples with SRH antibody titers above 85 mm^2^ increased significantly from 74.4% to 94.9% (*p*-value < 0.00001; Figure 3).

The diagnosis category had also significant differences with breeding and exportation categories (*p*-values = 0.0001 and <0.00001, respectively). Similarly to the sale category, a significant difference was measured between horses 2 years old or less and the others (*p*-value < 0.00001) (Figure 3).

Sixteen percent of serum samples from horses located in Pays de la Loire (*n* = 43) were below 85 mm^2^, including 10.4% samples with SRH antibody titer = 0 mm^2^. Of these samples, 30.5% were ≥85 mm^2^ and <154 mm^2^ (130.6 ± 19.5 mm^2^; *n* = 82) and 53.5% ≥154 mm^2^ (196.4 ± 28.3 mm^2^; *n* = 144). Overall, 84% of serum samples tested possessed SRH antibody titers above the clinical protection threshold (85 mm^2^). A significant difference was observed between the serum samples from horses located in this region and those based in Normandy and Auvergne-Rhône-Alpes (*p*-value = 0.027). The breeding category showed a frequency of serum samples with SRH antibody titers above 85 mm^2^ significantly higher (97.9%; *p*-value = 0.00081) compared to exportation, diagnosis and sale categories (63.8%, 80.6% and 77.8%, respectively). 

Nearly 9% (8.8%) of serum samples from horses located in Auvergne-Rhône-Alpes (*n* = 21) were below 85 mm^2^, including 4.2% samples with SRH antibody titer = 0 mm^2^. Of the samples, 31.3% were ≥85 mm^2^ and <154 mm^2^ (126.6 ± 20.6 mm^2^; *n* = 75) and 60.0% were ≥154 mm^2^ (198.3 ± 31.0 mm^2^; *n* = 144). Overall, 91.3% of serum samples tested possessed SRH antibody titers above the clinical protection threshold (85 mm^2^). A significant difference was observed between the serum samples from horses located in this region and those based in others (*p*-value = 0.016). No difference was measured between the two categories analyzed (*p*-value = 0.14). 

For serum samples from horses based in Occitanie, 13.7% of these samples (*n* = 41) were below 85 mm^2^, including 20 samples with SRH antibody titer = 0mm^2^ and 21 samples seroconverted (57.7 ± 20.2 mm^2^). 31.4% of samples were ≥85 mm^2^ and <154 mm^2^ (127.3 ± 18.9 mm^2^; *n* = 94) and 54.8% were ≥154 mm^2^ (186.9 ± 21.3 mm^2^; *n* = 164) (Figure 2). The serum samples from horses located in this region were significantly lower compared to the serum samples tested for the Normandy (*p*-value = 0.04). No significant difference has been shown between breeding and diagnosis categories. The frequency of serum samples, for both categories, with SRH antibody titers above the clinical protection threshold was 87%.

### 3.3. NP-ELISA Assays as DIVA (Differentiating Infected from Vaccinated Animals) Test

Four hundred and ten (410) serum samples were tested by the NP-ELISA assay. Forty-four (44) sera with an SRH antibody titer equal to 0 mm^2^ (13.9% of samples) were analyzed to confirm their seronegativity. Overall, 42 sera have been confirmed seronegative (95.5%) and 2 sera were seropositive (4.5%).

**Figure 4:**
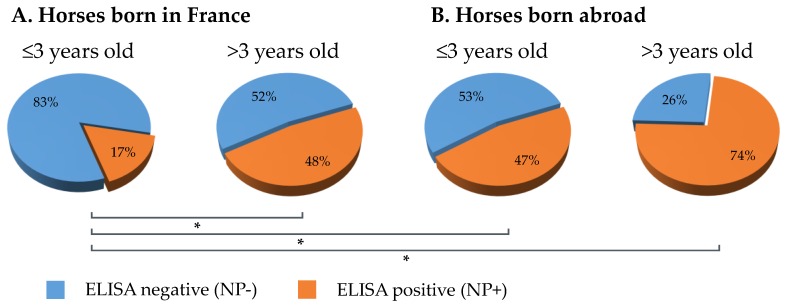
Comparison of equine influenza virus (EIV) nucleoprotein (NP) ELISA results between serum samples from young (≤3 years) and older (>3 years) horses at the time of sampling, which were seropositive by SRH test. Results for horses born in France (**A**) or abroad (**B**). (✻) *p*-value ≤ 0.006.

A comparison of EIV NP ELISA results between serum samples from young (≤3 years) and old (>3 years) horses (i.e., at the time of sampling) that were seropositive by SRH test was carried out. The ELISA results showed that 83% of serums (*n* = 136) from young horses born in France were NP-ELISA negative when compared with serums from old horses (*n* = 128) for which 52% were NP-ELISA negative. This difference was significant (*p*-value < 0.00001). A significant difference was also observed between serum samples from young horses born in France (*n* = 126) and abroad (*n* = 15; *p*-value = 0.006), the NP-ELISA negative for serum samples from horses born abroad was 53%. Only 26% of samples from old horses were negative by NP-ELISA (Figure 4).

Results were similar when analyzed by geographical regions, except for Auvergne-Rhône-Alpes were the number of samples available was too limited (data not shown).

### 3.4. 2018–2019 Equine Influenza Outbreaks, Phylogenetic Characterization and Relationship with Immune Status

#### 3.4.1. Equine Influenza Outbreak

**Figure 5:**
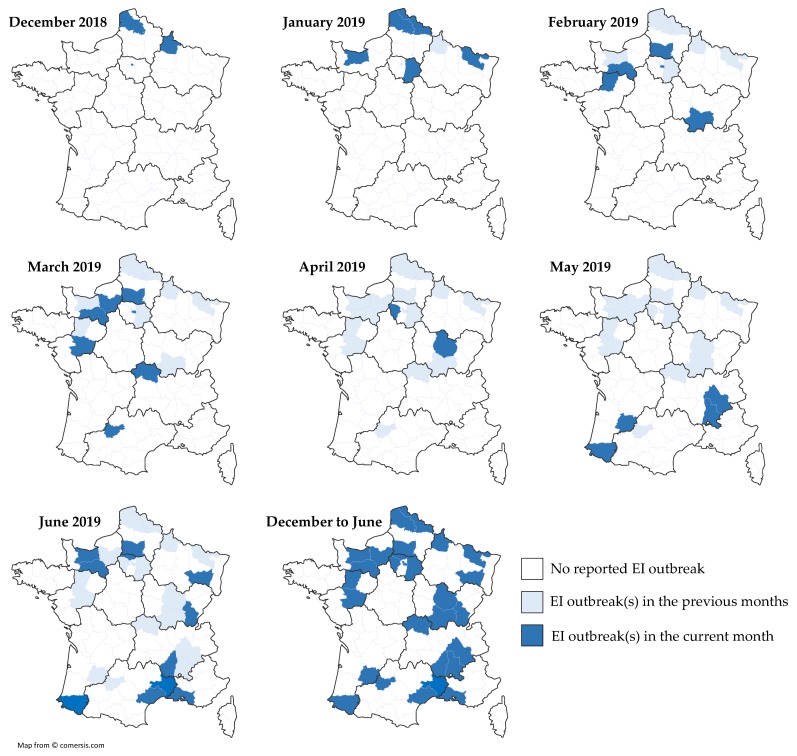
Localization of the French EI outbreaks at the county level, month by month. For each month, new EI cases are highlighted in dark blue, cases from previous months localized in the same county are highlighted in light blue.

From early December 2018 to the end of June 2019, 53 EI outbreaks were reported by the RESPE or diagnostic laboratories (Table 1). Regretfully, epidemiological information was not always available. Overall, the number of confirmed clinical cases were ≥246, from 29 different counties located in all French regions, with the exception of Britany and Corsica (Figure 5). 

#### 3.4.2. Phylogenetic Characterization

**Figure 6:**
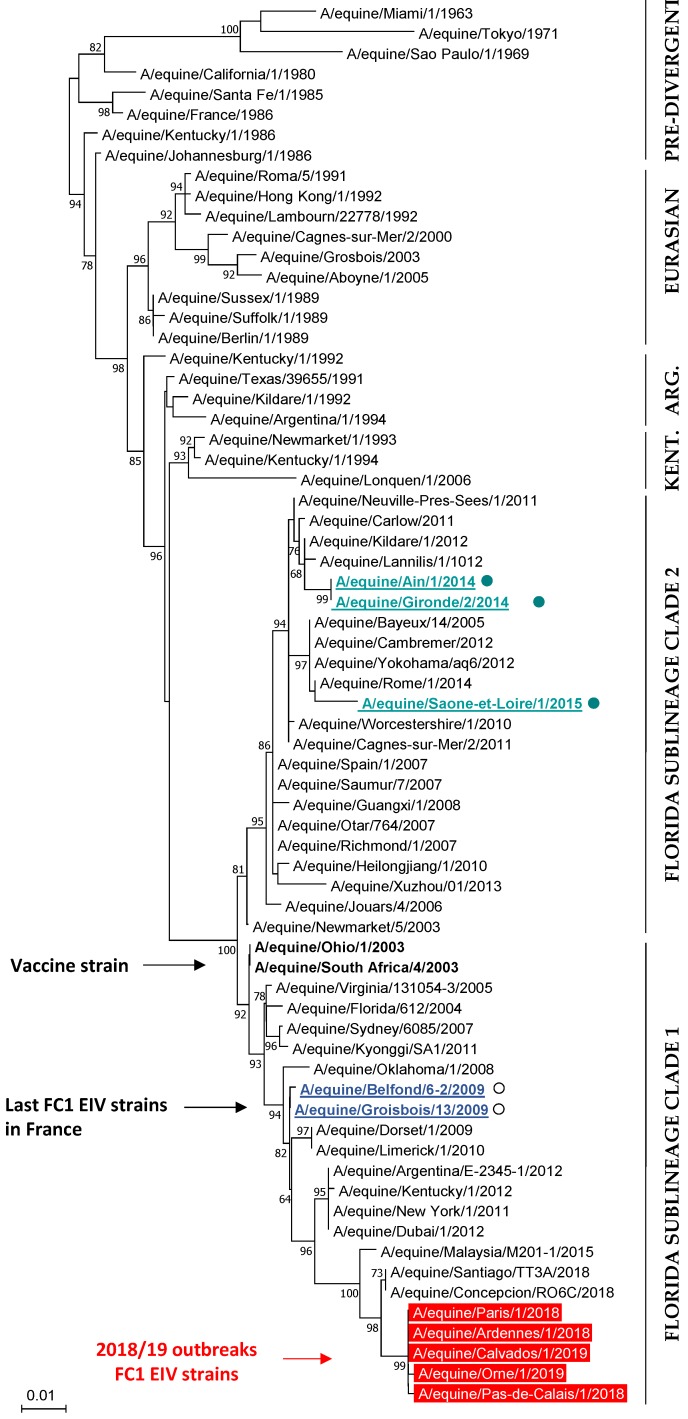
Phylogenetic analysis of the HA1 nucleotide sequence for 69 EIV strains, including representative strains of the main lineages and sub-lineages. The last French EIV strains belonging to the Florida sublineage clade 1 and clade 2 are underlined (open and closed circles, respectively). The FC1 EIV strains from the 2018 and 2019 French outbreaks are in red. The FC1 EIV vaccine strains are in bold text. Neighbor-Joining, Test phylogeny: Bootstrap method with 1000 bootstrap replication, Mode/method: Maximum composite Likelihood.

The HA genes from five EIV strains (EI outbreaks #1, #2, #3, #9 and #11) and the NA genes from two EIV strains (#2 and #9) were sequenced. Results reveals that H3N8 EIV strains at the origin of the 2018–2019 French outbreaks A/eq/Paris/1/2018 (MK501760), A/eq/Pas-de-Calais/1/2018 (MK501761 and MK501801), A/eq/Ardennes/1/2018 (MK501762), A/eq/Orne/1/2019 and A/eq/Calvados/1/2019 (MK501763 and MK501802) belong to the FC1 sub-lineage (Figure 6). HA gene sequencing reveals two amino-acid substitutions (p.T163I and p.A372T) when compared with FC1 EIV strains isolated in South America in 2018 (A/eq/Concepcion/RO6C/2018 and A/eq/Santiago/TT3A/2018), but 13 amino-acid substitutions with the closest FC1 EI vaccine strain A/eq/Ohio/03 (Supplementary Table S2), with four of them located in potential antigenic sites B (p.T163I and p.N188T) and E (p.R62K and p.N63D).

#### 3.4.3. 2018–2019 EI Outbreaks and EI Vaccination Status

The EI vaccination status at the time of infection was unknown for 134 horses. Vaccination status was known for 112 horses, with 68 (60.7%) declared as vaccinated against EI and 44 (39.3%) as unvaccinated (Table 1). Regretfully, details of EI vaccines used and the date of last vaccination were unknown for the majority of cases. Vaccination passports were available for 14 horses from 4 different outbreaks. As shown in Table 2, the time between infection/disease and last vaccination ranged from 1 month to 19.5 months. Thirteen out of fourteen horses received the recombinant canarypox-based HA vaccine (C) for their last EI immunization. All horses had annual or bi-annual boost immunizations prior to infection, with the exception of horse #4 that developed EI signs of disease 2 months after V3 and horse #7 that was infected 3.5 months after V2. Serum samples were obtained at the onset of clinical signs of diseases from 11 out of 14 horses, with 9 negatives by DIVA test, one providing a borderline result (#7) and one positive (#1), which indicates a possible seroconversion due to EIV infection or a reminiscent NP antibody response linked to a past immunization with a whole inactivated EI vaccine (W1), 17.5 months prior to sampling. SRH analyses shown that horse #7 had no measurable SRH antibody titer at the time of infection, which could be linked to the immunity gap frequently observed in the weeks preceding V3. Five out of eleven horses (#3, #4, #8, #9 and #13) had an SRH antibody titer below the clinical protection threshold (i.e., 85 mm^2^). Three horses (#1, #5 and #10) had an SRH antibody titer between the clinical protection threshold and the virological protection threshold (i.e., 154 mm^2^). One horse (#6) had an SRH antibody titer above the virological protection threshold at the time of infection.

**Figure 7:**
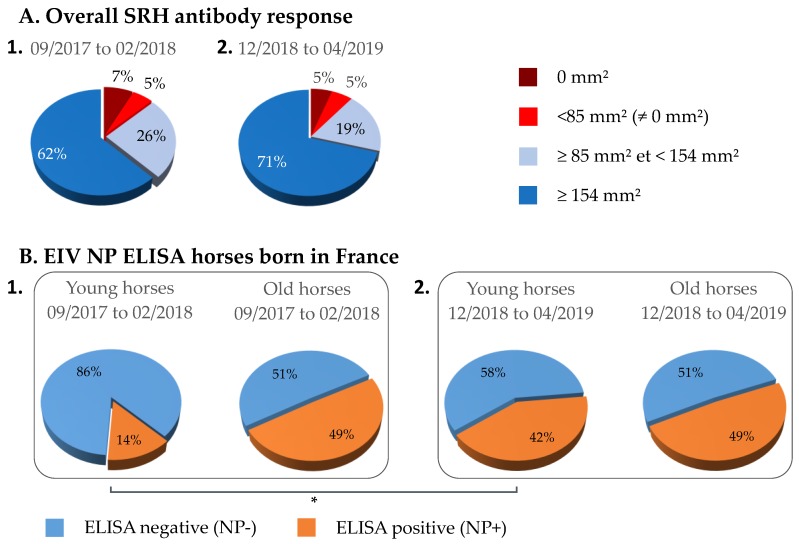
Comparison of overall SRH antibody response (**A**) and EIV NP ELISA (**B**) between two periods of sampling: September 2017 to February 2018 (1) and December 2018 to April 2019 (2). EIV NP ELISA results between serum samples from young (≤3 years; located in Normandy) and older (>3 years; located in the north of France) horses born in France and seropositive by SRH test. (✻) *p*-value ≤ 0.007.

Three hundred and fifty-nine (359) serum samples obtained from late December 2018 to April 2019 (time period 2) from horses located in the north of France were analyzed by SRH (Figure 7A) and compared with results from time period 1 (i.e., 2017–2018). A significant difference was measured between the two groups (*p*-value = 0.013) with an increased frequency of samples >154 mm^2^. Seventy-six (76) of these serum samples were analyzed with the EIV NP ELISA (DIVA test) in order to measure an eventual NP-specific antibody seroconversion. As illustrated in Figure 7B, the percent of SRH+ NP+ serums reached 42%, when compared with only 14% for serum samples obtained during time period 1 from horses located in Normandy (geographically close to the north of France) and ≤3 years old at the time of sampling (i.e., born after 2015; *p*-value = 0.0024), while it remained unchanged in older horses. While vaccination status is unknown and with the assumption that the type of EI vaccine used as not significantly changed, such increase tends to support circulation of EIV during this period of time.

## 4. Discussion

Recent data indicate that influenza vaccination coverage in the US is often below 60% in adult humans and below 45% in children [6]. This present study uses a serological test to evaluate the level of specific antibodies to EIV unlike equivalent studies in humans that favor questionnaires and epidemiological surveys. The overall analysis of immune coverage in horses from the four French regions studied highlights that only 12.4% of horses were considered as unprotected, based on their SRH antibody level at the time of sampling. A large proportion of horses have reached the virological protection threshold (60.3% ≥ 154 mm^2^). Overall immune coverage is estimated at 87.6%, a level described by Satou and Nishiura (2006) [12] as sufficient to protect an equine population against an equine influenza virus presenting a relatively low rate of transmission (retrospective study based on the 1971 EI outbreak in a Japanese racehorse facility). Such immune coverage in the French horse population studied could provide an explanation for a lack of detection of EIV and/or the disease between mid-2015 and late 2018, despite extensive surveillance by the RESPE and associated diagnostic laboratories (several hundreds of nasopharyngeal swab samples analyzed every year). Prior to this period, the last French EI outbreaks were reported in 2014 and 2015 but were limited in size and number (six and four, respectively) [13]. Other European countries with large equids population report EI cases every year [15,26]. While no or limited information is available, it would be interesting to investigate actual EI vaccine use and coverage in these countries to determine if more frequent and recurrent EI outbreaks recorded through the years may be explained by lower EI vaccination (e.g., anecdotal information based on the number of EI vaccine doses sold per year in relation to the number of horses may be used to calculate rough estimate of EI vaccine coverage).

This study, which aimed to provide an idea of the immune/vaccine coverage against EI in the French horse population, used a serological test to evaluate the level of EIV-specific antibodies unlike equivalent studies in humans or horses that used questionnaires, epidemiological survey and provide vaccine coverage results based on owners and veterinarians confirmation of vaccination. The SRH assay was selected for this study due to the well-known and described correlation between SRH antibody titers and protection against EIV infection, development of clinical signs of EI and virus shedding. The EIV strain used as SRH antigen was selected to be close to the FC2 recommended vaccine strain A/eq/Richmond/07, which is contained in the HA recombinant canarypox-based EI vaccine predominantly used in France. At the time of the serological study, EIV strains circulating in Europe were mostly belonging to the FC2 sub-lineage, which supported the SRH antigen choice. Cross-reactivity and cross-protection have also often been documented in recent vaccine clinical trials. Preliminary data indicates that serums used in this study also show cross-reactivity when tested against the FC1 EIV strain A/eq/Paris/1/2018, but further studies are warranted to explain the current EI epidemic in Europe. The current study aimed to be representative of the French horse population. However, some limitations were inevitable and should be taken into account for the interpretation of results: (i) four geographical regions that represented 57.5% of all French equids breeding centers were selected, with a known bias for Normandy due to the localization of the serum archive (i.e., LABÉO). When adjusted on the number of breeding centers per region (cf. Supplementary Figure S1), overall results were not significantly different (*p*-value = 0.085), with 86.8% above the 85 mm^2^ (when compared with 87.6% without adjustment, cf. Figure 1). (ii) Some horse populations are never visited by veterinary practitioners, for multiple reasons (e.g., economic, cultural, etc.). These populations, which are usually missed by the questionnaire and epidemiological surveys, are probably not represented in the current study. Obviously, such equids populations that are usually not vaccinated either, represent an important reservoir for pathogens and the weak link in any strategy of prevention.

Results for each French region selected in this study follow the same trend with protection rates reaching 84% to 87% (i.e., horses considered as vaccinated with clinical protection and reduced virus shedding in case of EIV infection). Some differences were measured between each sample’s categories. Results showed that “sale” and “diagnosis” categories were significantly lower when compared with other categories. Results indicate that after the exclusion of horses less than 2 years old at the time of sampling (born in 2016 and 2017), the immune coverage increased significantly from 79% to 96% for the “sale” category (in Normandy). A possible hypothesis is that horses born in 2016 and 2017 were sampled during their primary course of EI vaccination and may have not yet developed a complete and robust EI humoral immune response at the time of sampling. The results are correlated with a previous field study that highlighted a large frequency of Thoroughbred foals displaying low or negative SRH antibody titers during their primary EI immunization and up to 5 months after the third EI immunization (V3) [27] and other clinical studies highlighting the immunity gap frequently observed in the weeks preceding the third EI immunization [28,29,30]. In the field study, the frequency of seronegative foals (i.e., SRH = 0 mm^2^) was greater than 25% at different sampling time points during the primary EI immunization (at the time of first immunization, two weeks and three months after the second immunization and two days after the third immunization) [27]. After V3, the frequency of seronegative foals remained below 20% up to three months after this immunization [27]. In the present report, the frequency of horses located in Normandy and born in 2016 and 2017 with negative or low SRH titers was 49% and 42% for “sale” and “diagnosis” categories, respectively.

The categories “breeding” and “exportation” present high protection rates that reach 87% at least (excepted for the “exportation” category from Pays de la Loire). This observation is not surprising because these categories are submitted to mandatory EI vaccination in France. For the covering season of stallions, some studbooks require that EI vaccination be carried out in accordance with the EI vaccine manufacturers recommendations (i.e., primary vaccination with two immunizations 4–6 weeks apart and a third dose 5 to 6 months after the last immunization, then annual boost immunization afterward) otherwise breeding cannot be authorized. Similarly, for mares, studbooks and horses, breeding centers require EI vaccination. The regulation to export horses is also strict. In most cases, the sanitary measures imposed by importing countries require that horses have received two immunizations prior to movement. The information available does not provide an explanation for the higher percentage of negative and <85 mm^2^ samples in the “exportation” group for Pays de la Loire.

The use of an EIV NP-specific enzyme-linked immunosorbent assay (ELISA) allows differentiating infected animals from horses vaccinated with the HA recombinant canarypox-based EI vaccine (ProteqFlu^®^; Mérial, Lyon, France) [1]. Horses infected with EIV or vaccinated with a whole inactivated EI vaccine produce antibodies against all viral proteins, including the EIV NP. These horses have a positive serological status for the NP (NP+) unlike horses immunized with the recombinant canarypox EI vaccine that seroconvert to HA only and therefore have a negative serological status for the NP (NP−) [31]. The use of an EI vaccine with DIVA ability has proved very useful in past EI epidemics. Emergency vaccination was implemented during the 2007 Australian EI outbreak and this specific vaccine was selected for its successful use in South Africa in 2003 and its DIVA ability, amongst other reasons. The possibility to differentiate infected animals from vaccinates was of a great importance for the Australian’s EI outbreak management because it allowed to monitored EIV transmission and disease spread in light of ring EI vaccination [1]. The use of a DIVA assay to evaluate EI vaccination coverage in an endemic situation depends on the type of EI vaccine available and used in the field (i.e., recombinant or whole inactivated EI vaccines). In France, based on anecdotal discussions with EI vaccine manufacturers, the HA recombinant canarypox-based EI vaccine is predominantly used. In our current study, the use of a DIVA test confirmed that the EI seropositivity of young horses (≤3 years) was primarily due to EI vaccination using this specific EI vaccine. The DIVA analysis showed that 83% of samples from young horses born in France and seropositive by SRH assay were EIV NP-seronegative compared to old horses for which only 52% were EIV NP-seronegative. This observation is consistent with the last EI outbreak registered in 2015 (RESPE) and the predominant use of this specific HA recombinant EI vaccine in France. Before 2015, EI cases have been occasionally observed, which could explain higher frequency of older horses seropositive for EIV-NP (48%). The high level of young horses seronegative for EIV-NP supports the RESPE surveillance data that reports no EI clinical case in France between mid-2015 and late 2018. While the use of whole inactivated EI vaccines may explain EIV-NP seropositivity for the other 17% of young horses born in France, a low level of EIV circulation in France could not entirely be ruled out. Only 53% of young horses born abroad were EIV-NP seronegative. These results could be explained by a broader diversity of EI vaccines used and possible EIV circulation in other countries. The use of the DIVA test revealed that 96% horses with SRH antibody titers equal to 0 mm^2^ were also seronegative by NP-ELISA. In the absence of non-H3 equine influenza circulation, the remaining 4% (SRH negative and NP-ELISA) may be explained by a difference of sensitivity between the two assays. Hopefully, veterinary vaccine manufacturers will incorporate DIVA markers in their future EI vaccines, which will greatly benefit disease surveillance, would help to identify poor-vaccine responders and contribute to reducing fraudulent vaccination in horses in the long term (i.e., “pen vaccination”).

Despite high EIV-specific immune coverage and the use of an EI vaccine fully updated according to the last OIE recommendation on EI vaccine strain composition (i.e., EI vaccines should contain representative EIV strains of both FC1 and FC2 sub-lineages), the absence of clinical EI in France came to an end in early December 2019. Numerous EI outbreaks were reported in several European countries (Belgium, Germany, Ireland, the Netherlands, Sweden and the United Kingdom). The scale, number and duration of this epidemic had not been experienced in Europe since the late 1970s and 1980s. Sequencing results revealed that H3N8 EIV strains at the origin of the 2018–2019 French outbreaks (and EI outbreaks in other European countries, OIE ESP communication) belong to the FC1 sub-lineage, which was not isolated in France since 2009 [13] and was usually circulating in North and South Americas [17,20,32]. While several amino acids mutation were identified in the HA, results from the hemagglutination inhibition assay using mono-specific ferret sera and the associated antigenic cartography analyses indicate that FC1 EIV strains the origin of the 2018–2019 EI outbreaks were still antigenically closely related to the recommended FC1 EIV strains for inclusion in EI vaccine [33]. As a consequence, this epidemic was not considered by the OIE ESP to be linked to a mismatch with the EI vaccine strains and the EI vaccine strain recommendation remained unchanged in 2019. The introduction of more pathogenic strains could be an alternative explanation. The FC2 EIV strains isolated in Europe in recent years were of mild and decreasing pathogenicity [34], which may provide an explanation for the absence of EI in France in recent years (results from this study indicate that EI immunity/vaccine coverage is close to the level described by Satou and Nishiura [12]). However, a few anecdotal reports of EI induced mortality in the field in several EU countries and observation of mild but noticeable clinical signs of disease in EI vaccinated horses raise a question about the pathogenicity of the current FC1 EIV strain. At the time of this report, the current FC1 EIV strain has not been used in a controlled experimental infection, which prevents any strong assumption about its pathogenicity. Surveillance results reported in the current study highlight a larger amount of French EI outbreaks involving vaccinated horses, which is not entirely surprising when the high EI vaccine coverage measured here is taken into account. For the few cases where serum were obtained at the onset of disease, it appears that infection could be explained in half of the cases by a lower than expected SRH antibody titer at the time of contact with EIV (irrespective of the time since last immunization). With the exception of horse #6, which had an SRH antibody titer above the 154 mm^2^ threshold, other vaccinated horses had average titers (between 93 and 129 mm^2^), probably high enough to significantly reduce the clinical signs of disease but insufficient to induce sterilizing immunity, which is rarely measured [35], even in optimal study conditions.

The overall immune coverage in 2018, which was estimated at 87.6%, was sufficient to prevent EI clinical cases induced by FC2 EIV strains circulating in the EU between 2015 and late 2018. Regretfully, such an immune coverage (based on historical SRH protection thresholds) was not high enough to prevent the 2018–2019 FC1 EIV strains circulation. However, it is very important to note that all known field and veterinary reports indicate that clinical signs of disease observed in EI vaccinated horses were clearly reduced when compared with unvaccinated animals, which continue to support the benefit of EI vaccination. Vaccination has probably slowed the spread of EI in France, which provided invaluable time to Equine Veterinary Practitioners.

## 5. Conclusions

These results have a field significance for both equine veterinarians and the scientific community. When extrapolated to a country level, they highlight the success of EI vaccination and are concomitant with a lack of detection of the pathogen and an absence of clinical EI in the horse population in France. This information is particularly timely; the French horse population has suffered from an extended epizooty of equine herpesvirus infection in early 2018, which could be an indirect consequence of a nationwide equine herpesvirus vaccine shortage in 2016 and potential modification of vaccination habit and coverage against this specific pathogen in 2017. As a result, this study and results could inspire equine veterinary practitioners and horse owners to continue and intensify their effort in terms of vaccination. While EI immune/vaccine coverage and EI vaccines commercially available are challenged by this FC1 EIV strain, the benefit of EI immunization in mitigating the disease severity and to reduce transmission remains clear. Further work is warranted to explain the 2019 EI epidemic. This study also highlights the beneficial use of serology as a meaningful tool to support disease surveillance and management in horses, not only to evaluate the immune coverage at a country level but also in sensitive populations or prior to exportation in order to prevent entry of unprotected horses into a new country/population.

## Figures and Tables

**Table 1:** Details of French equine influenza (EI) outbreaks, from late 2018 to June 2019. Data were recorded by field equine veterinary practitioners and collated by the equine pathology epidemiological surveillance network (RESPE) and the LABÉO Research and Diagnostic Institute. Arab. = Arabian horse; Type = type of premises and/or breed; TB = thoroughbred; TC = training center; Horse/P = number of horses on the premises.

N	Date	Location		Type		Nb. Horses/P	Nb. of Clinical Cases	Vaccination Status
#1	14DEC18	Paris		Riding school		100	9	Yes
#2	21DEC18	Pas de Calais		French Saddlebred		50	14	No
#3	28DEC18	Ardennes		sport		unknown	3	No (young)
#4	02JAN19	Nord		sport		100	5	Yes
#5	10JAN19	Pas de Calais		Draft horse		40	1	No
#6	11JAN19	Pas de Calais		Belgian Warmblood, Connemara, French Saddlebred		300	2	Yes
#7	11JAN19	Pas de Calais		French Saddlebred		20	3	unknown
#8	11JAN19	Moselle		sport		130	3	unknown
#9	16JAN19	Calvados		unknown		unknown	2	unknown
#10	16JAN19	Seine et Marne		Zangersheide		40	1	yes
#11	07FEB19	Orne		French Trotters		50	12	yes
#12	13FEB19	Val de Marne		TC French Trotters		15	2	yes
#13	18FEB19	Saone et Loire		Paint horses, Arab.		15	5	yes
#14	20FEB19	Orne		Stud farm TB		unknown	2	yes (lapsed)
#15	20FEB19	Mayenne		TC Trotters		150	10	yes
#16	20FEB19	Val-de-Marne		TC Trotters		1	1	yes
#17	26FEB19	Oise		TC TB		12	10	unknown
#18	27FEB19	Oise		TC TB		unknown	2	unknown
#19	28FEB19	Val-de-Marne		TC Trotters		unknown	3	yes
#20	07MAR19	Orne		Trotter		unknown	1	yes
#21	07MAR19	Oise		TB		unknown	2 (no clin.)	unknown
#22	08MAR19	Val-de-Marne		Trotters		unknown	4	unknown
#23	09MAR19	Orne		TB		unknown	2	yes
#24	15MAR19	Oise		TB		unknown	2	unknown
#25	18MAR19	Val-de-Marne		Gelding		15	1	yes
#26	20MAR19	Orne		Trotters		70	63	unknown
#27	20MAR19	Eure		unknown		unknown	2	unknown
#28	20MAR19	Oise		TB		unknown	1	unknown
#29	20MAR19	Allier		unknown		unknown	1	unknown
#30	26MAR19	Maine et Loire		Trotters		unknown	1	unknown
#31	27MAR19	Allier		unknown		unknown	1	unknown
#32	29MAR19	Allier		TC TB		unknown	2	unknown
#33	29MAR19	Tarn et Garonne		unknown		unknown	1	unknown
#34	08APR19	Yvellines		unknown		unknown	2	unknown
#35	26APR19	Côte d’Or		Sport		unknown	9	unknown
#36	06MAY19	Isère		Leisure center		4	1	no
#37	17MAY19	Lot et Garonne		Sport Arab.		15	8	unknown
#38	24MAY19	Drôme		Breeding center, pony		8	7	no
#39	31MAY19	Pyrénées Atlantiques		Donkey		unknown	1	unknown
#40	01JUN19	Bouches du Rhône		unknown		unknown	1	unknown
#41	07JUN19	Jura		Heavy horse		15	15	no
#42	11JUN19	Hérault		unknown		unknown	1	unknown
#43	12JUN19	Vosges		Heavy horse		1	1	no
#44	12JUN19	Oise		unknown		unknown	unknown	unknown
#45	12JUN19	Val d’Oise		unknown		unknown	unknown	unknown
#46	12JUN19	Ardèche		Leisure center		30	4	yes
#47	12JUN19	Orne		French Trotters		28	10	yes
#48	19JUN19	Calvados		unknown		unknown	3	unknown
#49	22JUN19	Orne		unknown		unknown	1	unknown
#50	26JUN19	Orne		unknown		unknown	6	unknown
#51	27JUN19	Gard		Camargue		3	2	no
#52	28JUN19	Pyrénées Atlantiques		unknown		unknown	1 ^†^	unknown
#53	28JUN19	Orne		unknown		unknown	1	unknown
Number of counties/total ^1^			29/96		Total clinical cases		246	

^1^ total number of counties from mainland France. ^†^ animal found dead.

**Table 2:** EI clinical cases with known EI vaccination history: time since last vaccination and EIV-specific antibody responses (SRH and EIV NP ELISA) at the onset of clinical signs of disease: na = not applicable; nd = not done; neg = negative; pos = positive; V = last EI vaccine administered prior to EI outbreak; VT = vaccine type: C = EIV HA recombinant canarypoxvirus-based EI vaccine ProteqFlu (±Te), W1 = whole inactivated EI vaccine Equip FT, W2 = whole inactivated EI vaccine Equilis Prequenza; w = week.

Horse ID	Time Since Last Vaccination (V)		V-1/VT (Months between V and V-1)		V-2/VT (Months between V-1 and V-2)		SRH (mm^2^) ^1^	EIV NP ELISA DIVA	Outbreak
	Months	VT	Months	VT	Months	VT			
#1	1	C	9.5	C	7	W1	115.9 ^1^	pos	#1
#2	1	W1	12	C	8.5	C	nd	nd	#10
#3	2	C	6	C	6	C	70.6 ^1^	neg	#1
#4	2	C	6.5	C	1	C	74.05 ^1^	neg	#1
#5	3	C	2	C	12	C	105.9 ^1^	neg	#1
#6	3	C	11	C	6	C	177.2 ^2^	neg	#19
#7	3.5	C	1	C	na	na	0 ^1^	borderline	#1
#8	4	C	15	C	12	C	79.2 ^2^	neg	#19
#9	6	C	12	C	11.5	W2	66.9 ^1^	nd	#1
#10	9	C	12	C	1.5	C	129.5 ^1^	neg	#1
#11	9	C	11.5	C	6	C	93.9 ^2^	neg	#19
#12	9.5	C	12	C	12	C	nd	nd	#6
#13	11	C	12	C	7	C	45.6 ^1^	neg	#1
#14	19.5	C	12	C	12	C	nd	nd	#6

^1^ serum samples taken <3 days after EI confirmation by qRT-PCR. ^2^ serum samples taken on the day of EI confirmation by qRT-PCR.

## Supplementary Materials

The following are available online at https://www.mdpi.com/2076-393X/7/4/174/s1, Table S1: Details of serum samples analyzed and SRH antibody titers, by category, by regional origin. Table S2: HA amino acid alignment. Figure S1: Number of breeding centers per French region in 2017, all equids considered (race and sport horses, ponies, heavy horses and donkeys).
