# Supplementary material for: Success and Limitation of Equine Influenza Vaccination: The First Incursion in a Decade of a Florida Clade 1 Equine Influenza Virus that Shakes Protection Despite High Vaccine Coverage
Source: Vaccines (Basel). 2019 Nov 2;7(4):174. doi: 10.3390/vaccines7040174 (PMC6963532; doi:10.3390/vaccines7040174)

Success and limitation of equine influenza vaccination: the first incursion a decade of a Florida Clade 1 equine influenza virus that shakes protectio despite high vaccine coverage.

Stéphanie Fougerolle *et al*. Vaccines.

Supplementary Figure S1: Number of breeding centers per French region in 2017, all equids considered (race & sport horses, poneys, heavy horses and donkeys).

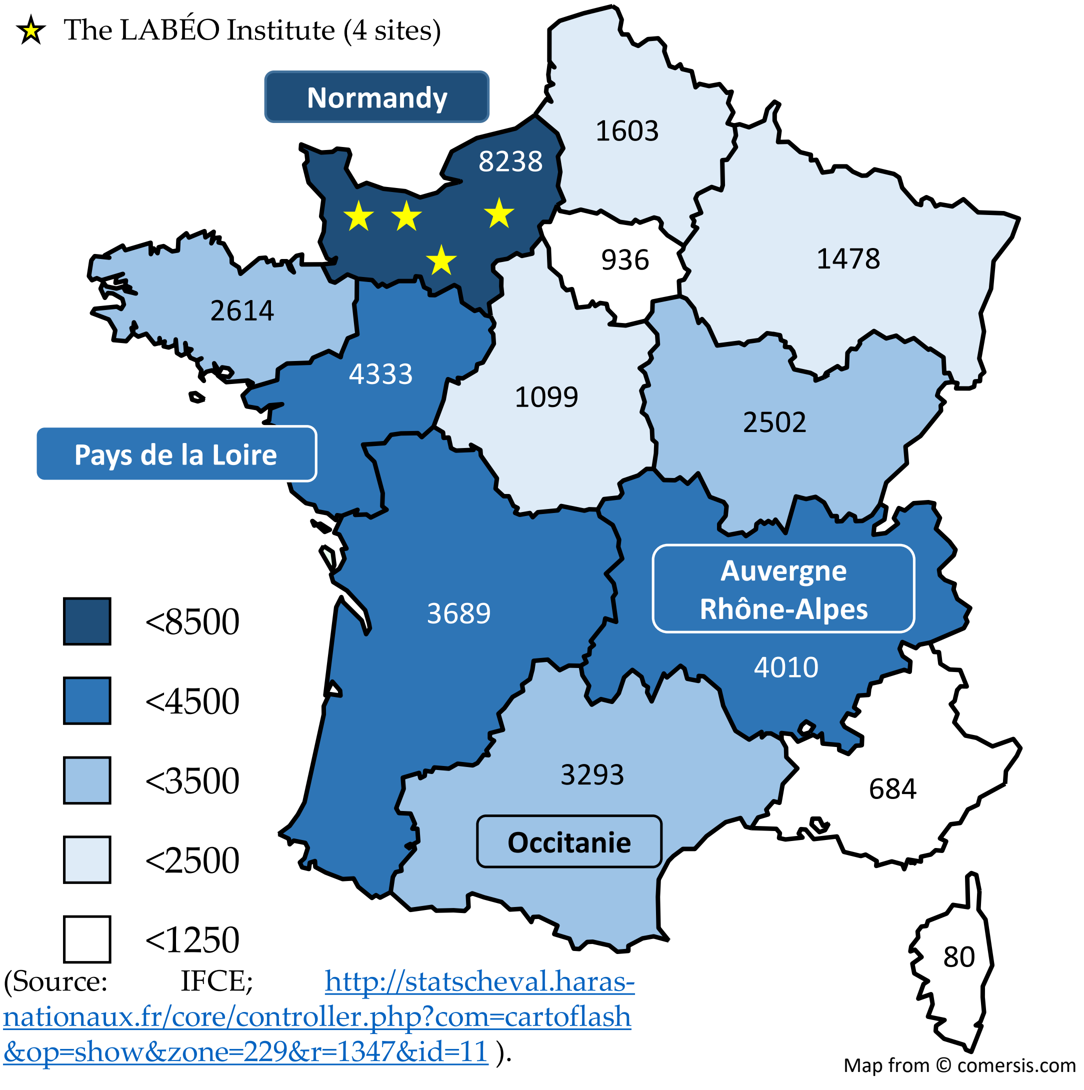

(Source: IFCE; http://statscheval.haras-nationaux.fr/core/controller.php?com=cartoflash&op=show&zone=229&r=1347&id=11).

SRH results adjusted to take into account the number of breeding center per region (i.e. to balance the weight of the Normandy region results).

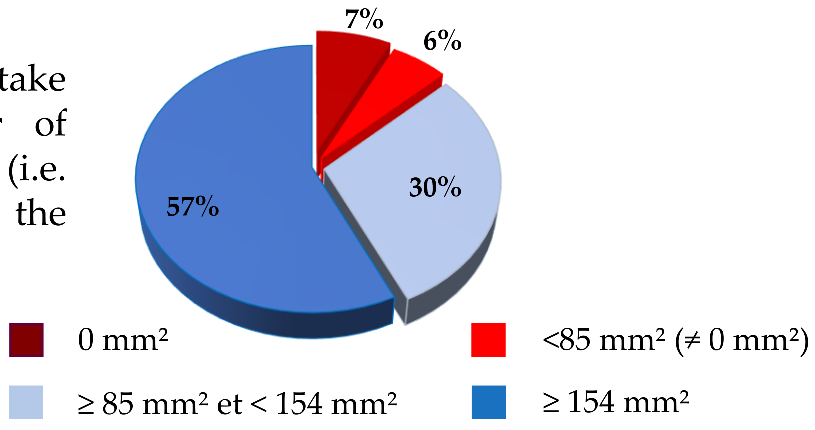

Supplement: Supplementary file 1 [file vaccines-07-00174-s001.zip › vaccines-622202 sup/vaccines-622202 author revision Sup Fig1.pdf]
