# Supplementary material for: Success and Limitation of Equine Influenza Vaccination: The First Incursion in a Decade of a Florida Clade 1 Equine Influenza Virus that Shakes Protection Despite High Vaccine Coverage
Source: Vaccines (Basel). 2019 Nov 2;7(4):174. doi: 10.3390/vaccines7040174 (PMC6963532; doi:10.3390/vaccines7040174)
Supplement: Supplementary file 1 [file vaccines-07-00174-s001.zip › vaccines-622202 sup/vaccines-622202 author revision Sup Table 1..pdf]

Success and limitation of equine influenza vaccination: the first incursion in a decade of a Florida Clade 1 equine influenza virus that shakes protection despite high vaccine coverage.

Stéphanie Fougerolle, Christine Fortier, Loïc Legrand, Marion Jourdan, Christel Marcillaud-Pitel, Stéphane Pronost and Romain Paillot.

Supplementary Table S1: SRH antibody titres of serum samples (collected between September 2017 to February 2018).

Region	Categories												Overall		
	Breeding			Exportation			Diagnosis			Sale					
	N	%	Average ± STDV (median)	N	%	Average ± STDV (median)	N	%	Average ± STDV (median)	N	%	Average ± STDV (median)	N	%	Average ± STDV (median)
Normandy	474			416			474			473			1837		
SRH=0mm^2	12	2.5		6	1.4		33	7		81	17.1		132	7.2	
SRH<85mm^2 ≠ 0mm^2	11	2.3	57.3 ± 15.5 (58.9)	9	2.1	48.3 ± 17.9 (46)	31	6.5	57.6 ± 18.8 (56.5)	40	8.5	56.8 ± 16.7 (56.1)	91	4.4	56.3 ± 17.4 (56.4)
SRH≥85mm^2 <155mm^2	115	24.3	132.9 ± 16.5 (135.7)	116	27.6	128.7 ± 19.9 (134.3)	116	24.5	126.3 ± 20.2 (128.4)	123	26	124.6 ± 17.4 (127.2)	470	25.6	128.2 ± 18.7 (131.3)
SRH≥155mm^2	336	70.9	194.7 ± 26.4 (191.7)	285	68.8	192.7 ± 28 (187.2)	294	62	197.8 ± 29 (193)	229	48.4	195.9 ± 30.3 (189.5)	1144	62.3	195.2 ± 28.3 (190.7)
Pays de Loire	95			36			93			45			269		
SRH=0mm^2	1	1.1		11	30.6		10	10.8		6	13.3		28	10.4	
SRH<85mm^2 ≠ 0mm^2	1	1.1	80.3 ± // (80.3)	2	5.6	53.7 ± 9.7 (53.7)	8	8.6	56.9 ± 10.9 (59.5)	4	8.9	50.2 ± 18.9 (49.1)	15	5.6	56.2 ± 14 (59.6)
SRH≥85mm^2 <155mm^2	32	33.7	135.9 ± 17.6 (139.7)	7	19.4	144.2 ± 7.3 (144.6)	27	29	126 ± 20.6 (126.7)	16	35.6	122 ± 19.8 (124.4)	82	30.5	130.6 ± 19.5 (135.9)
SRH≥155mm^2	61	64.2	193.1 ± 26.6 (185.8)	16	44.4	183.4 ± 22.5 (179.6)	48	51.6	202 ± 31.3 (201.4)	19	42.2	201.9 ± 26.5 (201.6)	144	53.5	196.4 ± 28.3 (191.4)

Rhône-Alpes	138			5			95			2			240		
SRH=0mm^2	3	2.2		0	0.0	/	6	6.3		1	50.0		10	4.2	
SRH<85mm^2 ≠ 0mm^2	6	4.3	70.4 ± 15.8 (76)	0	0.0	/	5	5.3	66.4 ± 11.3 (62.5)	0	0.0	/	11	4.6	68.6 ± 13.4 (68.7)
SRH≥85mm^2 <155mm^2	51	37	125.9 ± 20.3 (125.3)	0	0.0	/	24	25.3	127.9 ± 21.6 (136.1)	0	0.0	/	75	31.3	126.6 ± 20.6 (131.3)
SRH≥155mm^2	78	56.5	196.7 ± 31.9 (190.7)	5	100	212.1 ± 41.4 (195.3)	60	63.2	199.2 ± 29.3 (199.1)	1	50.0	194.3 ± // (194.3)	144	60.0	198.3 ± 31 (194.3)
Midi-Pyrénées	233			19			45			2			299		
SRH=0mm^2	20	5.6		5	26.3		2	4.4		0	0.0	/	20	6.7	
SRH<85mm^2 ≠ 0mm^2	21	6.9	60.5 ± 19 (69.4)	1	5.3	71.2 ± // (71.2)	4	8.9	43.2 ± 23 (36.7)	0	0.0	/	21	7.0	57.7 ± 20.2 (69.3)
SRH≥85mm^2 <155mm^2	94	33	127.6 ± 18.4 (131.9)	4	21.1	109.8 ± 23.4 (101)	12	26.7	132.7 ± 17.7 (133.5)	0	0.0	/	94	31.4	127.3 ± 18.9 (131.1)
SRH≥155mm^2	164	54.5	184.9 ± 20.5 (180.7)	9	47.4	190.6 ± 23.1 (190.9)	27	60.0	193 ± 23.4 (187.7)	2	100	211.5 ± 2.9 (211.5)	164	54.8	186.9 ± 21.3 (183)
Overall	940			476			707			522			2645		
SRH=0mm^2	29	3.1		22	4.6		51	7.2		88	16.9		190	7.2	
SRH<85mm^2 ≠ 0mm^2	34	3.6	61.8 ± 17.5 (64.2)	12	2.5	51.1 ± 16.9 (50.7)	48	6.8	57.2 ± 17.7 (58.3)	44	8.4	56.2 ± 16.8 (56.1)	138	5.2	57.5 ± 17.4 (58.8)
SRH≥85mm^2 <155mm^2	275	25.6	130.5 ± 18.2 (134.6)	128	25.6	128.8 ± 20.1 (135.3)	179	22.1	126.9 ± 20.2 (130.1)	139	23.2	124.3 ± 17.6 (127.1)	721	27.3	128.1 ± 19.1 (131.5)
SRH≥155mm^2	602	64	192.7 ± 26.4 (188.5)	314	66.5	192.5 ± 27.9 (187.8)	429	60.7	198.3 ± 29 (194.3)	251	48.1	196.5 ± 29.9 (191.4)	1596	60.3	194.8 ± 28 (190.2)
