# Supplementary material for: Success and Limitation of Equine Influenza Vaccination: The First Incursion in a Decade of a Florida Clade 1 Equine Influenza Virus that Shakes Protection Despite High Vaccine Coverage
Source: Vaccines (Basel). 2019 Nov 2;7(4):174. doi: 10.3390/vaccines7040174 (PMC6963532; doi:10.3390/vaccines7040174)
Supplement: Supplementary file 1 [file vaccines-07-00174-s001.zip › vaccines-622202 sup/vaccines-622202 author revision Sup Table 2..pdf]

Success and limitation of equine influenza vaccination: the first incursion in a decade of a Florida Clade 1 equine influenza virus that shakes protection despite high vaccine coverage.
Stéphanie Fougerolle *et al*. Vaccines.

Supplementary Table S2: HA amino acid alignment.

```
                           1   .   10    .   20    .   30    .   40    .   50    .   60    .   70    .   80    .   90    .  100
A/eq/Ohio/1/2003           SQNPISGNNTATLCLGHHAVANGTLVKTISDDQIEVTNATELVQSISMGKICNNSYRILDGRNCTLIDAMLGDPHCDAFQYENWDLFIERSSAFSNCYPY
A/eq/South Africa/4/2003   ....................................................................................................
A/eq/East Lothian/1/2018   .....ND.......................................P..............KD.....................................
A/eq/Santiago/TT3A/2018    .....ND.......................................P..............KD.....................................
A/eq/Concepcion/RO6C/2018  .....ND.......................................P..............KD.....................................
A/eq/Paris/1/2018          .....ND.......................................P..............KD.....................................
A/eq/Pas-de-Calais/1/2018  .....ND.......................................P..............KD.....................................
A/eq/Ardennes/1/2018       .....ND.......................................P..............KD.....................................
A/eq/Calvados/1/2019       .....ND.......................................P..............KD.....................................
A/eq/Orne/1/2019           .....ND.......................................P..............KD.....................................
                           101 .  110    .  120    .  130    .  140    .  150    .  160    .  170    .  180    .  190    .  200
A/eq/Ohio/1/2003           DIPDYASLRSIVASSGTLEFTAEGFTWTGVTQNGRSGACKRGSADSFFSRLNWLTKSGSSYPTLNVTMPNNKNFDKLYIWGIHHPSSNQEQTKLYIQESG
A/eq/South Africa/4/2003   ....................................................................................................
A/eq/East Lothian/1/2018   ...N.................................S.................................................T............
A/eq/Santiago/TT3A/2018    ...N.................................S.................................................T............
A/eq/Concepcion/RO6C/2018  ...N.................................S.................................................T............
A/eq/Paris/1/2018          ...N.................................S........................I........................T............
A/eq/Pas-de-Calais/1/2018  ...N.................................S........................I........................T............
A/eq/Ardennes/1/2018       ...N.................................S........................I........................T............
A/eq/Calvados/1/2019       ...N.................................S........................I........................T............
A/eq/Orne/1/2019           ...N.................................S........................I........................T............
                           201 .  210    .  220    .  230    .  240    .  250    .  260    .  270    .  280    .  290    .  300
A/eq/Ohio/1/2003           RVTVSTKRSQQTIIPNIGSRPWVRGQSGRISIYWTIVKPGDILMINSNGNLVAPRGYFKLKTGKSSVMRSDVPIDICVSECITPNGSISNDKPFQNVNKV
A/eq/South Africa/4/2003   ....................................................................................................
A/eq/East Lothian/1/2018   ......................I.............................................................................
A/eq/Santiago/TT3A/2018    ......................I.............................................................................
A/eq/Concepcion/RO6C/2018  ......................I.............................................................................
A/eq/Paris/1/2018          ......................I.............................................................................
A/eq/Pas-de-Calais/1/2018  ......................I.............................................................................
A/eq/Ardennes/1/2018       ......................I.............................................................................
A/eq/Calvados/1/2019       ......................I.............................................................................
A/eq/Orne/1/2019           ......................I.............................................................................
                           301 .  310    .  320    .  330    .  340    .  350    .  360    .  370    .  380    .  390    .  400
A/eq/Ohio/1/2003           TYGKCPKYIRQNTLKLATGMRNVPEKQIRGIFGAIAGFIENGWEGMVDGWYGFRYQNSEGTGQAADLKSTQAAIDQINGKLNRVIERTNEKFHQIEKEFS
A/eq/South Africa/4/2003   .................................-------------------------------------------------------------------
A/eq/East Lothian/1/2018   ....................................................................................................
A/eq/Santiago/TT3A/2018    ....................................................................................................
A/eq/Concepcion/RO6C/2018  ....................................................................................................
A/eq/Paris/1/2018          .......................................................................T............................
A/eq/Pas-de-Calais/1/2018  .......................................................................T............................
A/eq/Ardennes/1/2018       .......................................................................T............................
A/eq/Calvados/1/2019       .......................................................................T............................
A/eq/Orne/1/2019           .......................................................................T............................
                           401 .  410    .  420    .  430    .  440    .  450    .  460    .  470    .  480    .  490    .  500
A/eq/Ohio/1/2003           EVEGRIQDLEKYVEDTKIDLWSYNAELLVALENQHTIDLTDAEMNKLFEKTRRQLRENAEDMGGGCFKIYHKCDNACIGSIRNGTYDHYIYRDEALNNRF
A/eq/South Africa/4/2003   ----------------------------------------------------------------------------------------------------
A/eq/East Lothian/1/2018   .................................................R...L...................N..........................
A/eq/Santiago/TT3A/2018    .................................................G...L...................N..........................
A/eq/Concepcion/RO6C/2018  .................................................R...L...................N..........................
A/eq/Paris/1/2018          .................................................R...L...................N..........................
A/eq/Pas-de-Calais/1/2018  .................................................R...L...................N..........................
A/eq/Ardennes/1/2018       .................................................R...L...................N..........................
A/eq/Calvados/1/2019       .................................................R...L...................N..........................
A/eq/Orne/1/2019           .................................................R...L...................N..........................
                           501 .  510    .  520    .  530    .  540    .  550
A/eq/Ohio/1/2003           QIKGVELKSGYKDWILWISFAISCFLICVVLLGFIMWACQKGNIRCNICI
A/eq/South Africa/4/2003   --------------------------------------------------
A/eq/East Lothian/1/2018   ..................................................
A/eq/Santiago/TT3A/2018    ..................................................
A/eq/Concepcion/RO6C/2018  ..................................................
A/eq/Paris/1/2018          ..................................................
A/eq/Pas-de-Calais/1/2018  ..................................................
A/eq/Ardennes/1/2018       ..................................................
A/eq/Calvados/1/2019       ..................................................
A/eq/Orne/1/2019           ..................................................
```
